# Zika Virus IgM Detection and Neutralizing Antibody Profiles 12–19 Months after Illness Onset 

**DOI:** 10.3201/eid2502.181286

**Published:** 2019-02

**Authors:** Isabel Griffin, Stacey W. Martin, Marc Fischer, Trudy V. Chambers, Olga Kosoy, Alyssa Falise, Olga Ponomareva, Leah D. Gillis, Carina Blackmore, Reynald Jean

**Affiliations:** Florida Department of Health in Miami–Dade County, Miami, Florida, USA (I. Griffin, A. Falise, O. Ponomareva, R. Jean);; Centers for Disease Control and Prevention, Fort Collins, Colorado, USA (S.W. Martin, M. Fischer, T.V. Chambers, O. Kosoy);; Bureau of Public Health Laboratories, Miami (L.D. Gillis);; Florida Department of Health, Tallahassee, Florida, USA (C. Blackmore)

**Keywords:** Zika virus, immunoglobulin, IgM, MAC-ELISA, flaviviruses, neutralizing antibodies, viruses, Florida, United States

## Abstract

Data on the duration of detectable Zika virus–specific IgM in infected persons are limited. Neutralizing antibody cross-reactivity occurs between Zika virus and related flaviviruses, but the degree to which this confounds diagnosis is uncertain. We tested serum specimens collected 12–19 months after illness onset from patients with confirmed Zika virus disease for Zika virus IgM and Zika virus and dengue virus neutralizing antibodies. Among 62 participants, 45 (73%) had detectable Zika virus IgM and 12 (19%) had an equivocal result. Although all patients tested had Zika virus neutralizing antibodies, 39 (63%) also had neutralizing antibodies against dengue virus; of those, 12 (19%) had <4-fold difference between Zika virus and dengue virus titers, and 5 (8%) had dengue virus titer >4-fold higher than Zika virus titer. Prolonged detection of IgM and neutralizing antibody cross-reactivity make it difficult to determine the timing of Zika virus infection and differentiate between related flaviviruses.

Zika virus is a flavivirus closely related to dengue, West Nile, Japanese encephalitis, and yellow fever viruses (*1*,*2*). Diagnostic testing for Zika virus infection is conducted using both molecular and serologic methods, which include testing for viral RNA and IgM and neutralizing antibodies (*3*–*5*). RNA detection is most sensitive during the acute phase of illness and confirms Zika virus infection, but sensitivity declines after the first week of illness and a negative result does not exclude infection. Zika virus IgM typically develops <4 days after symptom onset and remain detectable for at least 12 weeks (*6*–*8*). Data on the duration of IgM after Zika virus infection are lacking, but IgM against other flaviviruses can last for months to years following infection (*9*–*13*). Neutralizing antibodies develop shortly after IgM, persist for many years, and may confer lifelong immunity (*13*,*14*).

Cross-reactivity between Zika virus and other flaviviruses occurs both with IgM and neutralizing antibodies and makes distinguishing Zika virus from dengue virus infections especially challenging. Whereas primary Zika virus infections typically generate highly specific neutralizing antibodies, secondary flavivirus infections show a high degree of cross-reactivity (*6*,*15*,*16*). For secondary infections, it remains uncertain whether the infecting flavivirus neutralizing antibody response is significantly greater than the cross-reacting neutralizing response, allowing for differentiation, and whether cross-reactive neutralizing antibodies are maintained for months to years after infection (*16*–*19*).

In July 2016, the first Zika virus outbreak in the continental United States was identified in Florida, culminating in 300 locally acquired cases in 2016 (*20*,*21*). We collected serum specimens from patients with Zika virus infection confirmed by molecular testing to determine the proportion of patients with detectable Zika virus IgM and the ratio of Zika virus and dengue virus neutralizing antibodies at 12–19 months after their acute illness.

## Methods

Eligible participants were residents of Miami–Dade County, Florida, USA, who had Zika virus disease confirmed by real-time reverse transcription PCR (rRT-PCR) and symptom onset during June–October 2016. Persons with asymptomatic infection, pregnant women, and infants with congenital infection were excluded from enrollment. We enrolled participants during October 16, 2017–February 1, 2018. We obtained written consent from study participants or their guardians.

Serum specimens were tested at the Centers for Disease Control and Prevention (Fort Collins, CO, USA) by IgM antibody capture ELISA (MAC-ELISA) for detection of Zika virus and dengue virus IgM and by plaque reduction neutralization test (PRNT) to detect Zika virus and dengue virus neutralizing antibodies (*5*,*6*,*22*). The PRNT endpoint titer was defined as the reciprocal of the dilution reducing the virus plaque count by 90%.

We obtained descriptive and clinical data for case-patients, including age, gender, race/ethnicity, reported symptoms, symptom onset, and origin of infection, from Merlin, the Florida Department of Health surveillance system. We used Pearson χ^2^ and Fisher exact tests to examine associations between demographics, symptomology, and Zika virus IgM results. We performed all statistical analyses with SAS statistical software version 9.4 (https://www.sas.com/en_us/software/sas9.html*)*. This study was approved by the Florida Department of Health Institutional Review Board.

## Results

Of 352 eligible PCR-confirmed Zika virus disease case-patients, 62 (18%) were enrolled and provided follow-up serum specimens. The 62 enrolled participants and 290 eligible case-patients who were not enrolled were similar with regard to age, sex, race/ethnicity, and clinical manifestations; however, 55% of enrolled participants acquired their infections in Florida, compared with 45% of the unenrolled cases (Table 1).

**Table 1:** Demographic and clinical characteristics of confirmed Zika virus disease case-patients in Miami–Dade County, Florida, USA

Characteristic	No. (%) enrolled,* n = 62	No. (%) not enrolled,* n = 290	p value†
Age group, y			0.19
1–18	2 (3)	26 (9)	
19–64	54 (87)	247 (85)	
>65	6 (10)	17 (6)	
Sex			0.85
M	32 (52)	146 (50)	
F	30 (48)	144 (50)	
Race/ethnicity			0.55
Non-Hispanic white	13 (21)	41 (14)	
Non-Hispanic African American	3 (5)	14 (5)	
Hispanic	42 (68)	209 (72)	
Unknown	4 (6)	26 (9)	
Main symptoms of Zika virus disease‡			0.88
1 of 4	2 (3)	12 (4)	
2 of 4	15 (24)	78 (27)	
3 of 4	32 (52)	130 (45)	
4 of 4	13 (21)	69 (24)	
Origin of infection			0.19
Florida	34 (55)	133 (46)	
Outside Florida	28 (45)	157 (54)	

*All percentages are column percentages. †A p value <0.05 was considered statistically significant. ‡Main symptoms were defined as fever, maculopapular rash, arthralgia, and conjunctivitis.

Among the enrolled participants, 8 (13%) provided a specimen at 12 months after initial symptom onset, 1 (2%) at 13 months, 13 (21%) at 14 months, 21 (34%) at 15 months, 11 (18%) at 16 months, 3 (5%) at 17 months, 3 (5%) at 18 months, and 2 (3%) at 19 months. The median age of participants was 47 years (range 8–70 years); 60 (97%) were adults >18 years of age (Table 1). Overall, 32 (52%) participants were male, and 42 (68%) were Hispanic. Two (3%) participants reported only 1 of the 4 main symptoms (fever, maculopapular rash, arthralgia, and conjunctivitis) at the time of their initial Zika virus diagnosis; 15 (24%) reported 2, 32 (52%) reported 3, and 13 (21%) reported all 4. From case investigations, we determined that 34 (55%) participants acquired their Zika virus infection locally in Miami–Dade County.

At follow-up, 45 (73%) patients had detectable Zika virus IgM, 12 (19%) had an equivocal result, and 5 (8%) were negative (all laboratory results provided in Appendix). Results by month since Zika virus symptom onset (Figure) showed that, overall, 39 (91%) of 43 specimens collected at 12–15 months postonset were IgM positive or equivocal, and 18 (95%) of 19 specimens collected at 16–19 months were positive or equivocal. No significant differences in IgM persistence were identified by age, gender, race/ethnicity, origin of infection, or time since illness onset (Table 2).

**Figure:**
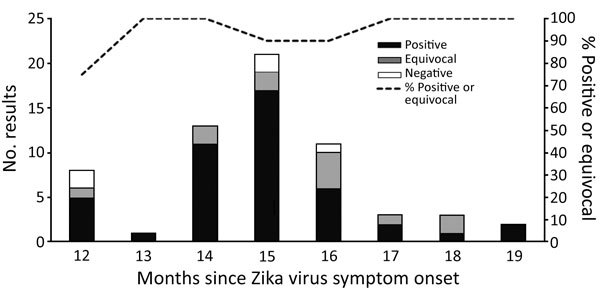
Zika virus IgM results for 62 participants in Miami–Dade County, Florida, USA, with PCR-confirmed Zika virus disease by follow-up specimen collection month.

**Table 2:** Demographic and clinical characteristics of enrolled participants in Miami–Dade County, Florida, USA, with PCR-confirmed Zika virus disease by Zika virus IgM antibody result 12–19 months after illness onset (n = 62)

Characteristic	Zika virus IgM results 12–19 mo after symptom onset, no. (%)*			p value†
	Positive	Equivocal	Negative	
Age group, y				0.58
1–18, n = 2	2 (100)	0	0	
19–64, n = 54	38 (71)	12 (22)	4 (7)	
>65, n = 6	5 (83)	0	1 (17)	
Sex				0.30
M, n = 32	25 (78)	6 (19)	1 (3)	
F, n = 30	20 (67)	6 (20)	4 (13)	
Race/ethnicity				0.18
Non-Hispanic white, n = 12	11 (92)	1 (8)	0	
Non-Hispanic African American, n = 4	1 (25)	2 (50)	1 (25)	
Hispanic, n = 42	29 (69)	9 (21)	4 (10)	
Unknown, n = 4	4 (100)	0	0	
Main symptoms of Zika virus‡				0.28
1 of 4, n = 2	1 (50)	1 (50)	0	
2 of 4, n = 15	8 (54)	5 (33)	2 (13)	
3 of 4, n = 32	25 (78)	4 (13)	3 (9)	
4 of 4, n = 13	11 (85)	2 (15)	0	
Origin of infection				0.52
Florida, n = 34	24 (70)	6 (18)	4 (12)	
Outside Florida, n = 28	21 (75)	6 (21)	1 (4)	

*All percentages are row percentages. †A p-value <0.05 was considered statistically significant. ‡Main symptoms were defined as fever, maculopapular rash, arthralgia, and conjunctivitis.

All participants had Zika virus neutralizing antibodies at 12–19 months after their acute illness, and 39 (63%) had dengue virus neutralizing antibody titers at follow-up. Using a definition of positive or equivocal Zika virus IgM with confirmatory Zika virus neutralizing antibodies, 57 (92%) would have had a diagnosis of recent Zika virus or flavivirus infection on the basis of results from their follow-up specimens (Table 3). Overall, regardless of Zika virus IgM results, 45 (73%) of the PCR-confirmed cases had Zika virus neutralizing antibody titers that were >4-fold higher than dengue virus titers. However, substantial cross-reactivity in neutralizing antibodies was still observed; 12 (19%) patients had a <4-fold difference between Zika virus and dengue virus titers, and 5 (8%) had dengue virus titers that were >4-fold higher than Zika virus titers.

**Table 3:** Diagnostic test interpretations among participants with PCR-confirmed Zika virus disease in Miami–Dade County, Florida, USA, based on IgM and neutralizing antibody results 12–19 months after onset*

Test results	No. (%), n = 62	Interpretation†
Zika virus IgM positive or equivocal, n = 57		
Zika virus PRNT titer >10 and DENV PRNT titer <10	21 (34)	Recent Zika virus infection
Zika virus PRNT titer >4-fold higher than DENV PRNT titer	21 (34)	Recent flavivirus infection
<4-fold difference between Zika virus and DENV PRNT titers	11 (18)	Recent flavivirus infection
DENV PRNT titer >4-fold higher than Zika virus PRNT titer	4 (6)	Recent flavivirus infection
ZIKV IgM negative, n = 5		
Zika virus PRNT titer >10 and Zika virus PRNT titer <10	2 (3)	Previous Zika virus infection
Zika virus PRNT titer >4-fold higher than DENV PRNT titer	1 (2)	Previous flavivirus infection
<4-fold difference between Zika virus and DENV PRNT titers	1 (2)	Previous flavivirus infection
DENV PRNT titer >4-fold higher than Zika virus PRNT titer	1 (2)	Previous flavivirus infection

*DENV, dengue virus; PRNT, plaque reduction neutralization test. †Recent Zika virus infection: Zika virus IgM positive or equivocal with a Zika virus PRNT titer ≥10 and dengue PRNT titer <10; recent flavivirus infection: Zika virus IgM positive or equivocal with a Zika virus PRNT titer >10 or dengue PRNT titer >10; previous Zika virus infection: Zika virus IgM negative with a Zika virus PRNT titer ≥10 and dengue PRNT titer <10; previous flavivirus infection: Zika virus IgM negative with a Zika virus PRNT titer >10 or dengue PRNT titer >10.

## Discussion

These findings demonstrate that 73% of persons with PCR-confirmed symptomatic Zika virus disease still had positive IgM test results 12–19 months after their initial illness, and another 19% had equivocal results. Because all participants had Zika virus neutralizing antibodies, a high proportion (92%) would have had a recent Zika virus or flavivirus infection diagnosis on the basis of serologic testing performed at the follow-up time point. Current Zika virus testing guidance recommends Zika virus serologic diagnosis only for symptomatic patients with a clinically compatible Zika virus illness (*3*). However, given the limited specificity of the clinical symptoms associated with Zika virus disease, the prolonged detection of Zika virus IgM presents a particular challenge for serologic diagnosis in pregnant women, given the importance of determining if the infection occurred during the current pregnancy, and complicates the diagnosis of new Zika virus infections in locations with known previous outbreaks.

The prolonged detection of IgM after Zika virus infection is consistent with previous findings for related flaviviruses (*9*–*13*). In a study of patients with confirmed West Nile virus encephalitis, 9 (43%) of 21 patients had detectable IgM 300–400 days after onset of their acute illness, and 5 (42%) of 12 had IgM detected >500 days after onset (*11*). Among asymptomatic blood donors with West Nile virus viremia detected on routine screening, IgM was still detected an average of 156 days (95% CI 70–423 days) after the viremic donation (*13*). A similar finding was observed in a study looking at the immune response to yellow fever vaccine in adults, in which 29 (73%) of 40 persons had detectable levels of IgM 3–4 years postvaccination (*12*).

Serologic cross-reactivity has been demonstrated between Zika virus and related flaviviruses, including dengue virus, but the degree to which neutralizing antibody cross-reactivity limits the ability to identify the specific virus responsible for the current infection is unclear (*6*,*23*). One published report suggests that relative levels of neutralizing antibody titers can distinguish Zika virus from dengue virus infections, especially in specimens collected months after infection (*19*). However, we found substantial neutralizing antibody cross-reactivity in >25% of specimens collected >1 year after symptom onset.

These findings are subject to several limitations. This report presents data from a single follow-up specimen but how long IgM may persist after this timeframe remains unknown. We cannot exclude the possibility that some participants may have been reexposed to Zika virus or another flavivirus between their initial illness and follow-up testing. The timing of the follow-up specimen varied among the participants and was limited to 12–19 months following onset of Zika virus symptoms. This, coupled with the small number of specimens at some time points, prevented us from assessing possible trends in IgM persistence over time. The small sample size and lack of specimens from the acute illness also limited our ability to detect factors that may be associated with prolonged detection of IgM, including possible differences between primary and secondary infections. Our findings of prolonged IgM seropositivity are specific to the Centers for Disease Control and Prevention MAC-ELISA, which targets Zika virus premembrane and envelope glycoproteins; other IgM serologic assays targeting other proteins are currently available and may not produce comparable findings. Finally, because we enrolled only symptomatic disease case-patients, it is uncertain whether persons with asymptomatic infections would exhibit similar IgM persistence.

These findings support data for other flaviviruses and suggest that a substantial proportion of persons with Zika virus disease will still have detectable IgM 1–2 years after their initial infection. The results highlight the complexity of using serologic diagnosis to determine the specific timing of a recent infection, which is particularly important for pregnant women and challenging for residents of areas with previous or ongoing Zika virus activity. As such, the findings further support the current recommendations to use nucleic acid amplification for screening asymptomatic pregnant women with ongoing possible Zika virus exposure (*3*). Further study is needed to assess IgM persistence using other approved assays, determine the full duration of Zika virus IgM after infection, and evaluate possible differences in IgM duration following primary and secondary infections.

Appendix Diagnostic test results for participants with PCR-confirmed Zika virus disease in Miami–Dade County, Florida, USA, 12–19 months after onset.
