# Supplementary material for: Zika Virus IgM Detection and Neutralizing Antibody Profiles 12–19 Months after Illness Onset
Source: Emerg Infect Dis. 2019 Feb;25(2):299–303. doi: 10.3201/eid2502.181286 (PMC6346474; doi:10.3201/eid2502.181286)
Supplement: Appendix — Diagnostic test results for participants with PCR-confirmed Zika virus disease in Miami–Dade County, Florida, USA, 12–19 months after onset. [file 18-1286-Techapp-s1.pdf]

Article DOI: https://doi.org/10.3201/eid2502.181286

Zika Virus IgM Detection and Neutralizing Antibody Profiles 12–19 Months after Illness Onset

Appendix

Appendix Table. Diagnostic test results among participants with PCR-confirmed Zika virus disease in Miami-Dade County, Florida, USA, 12–19 months after onset (n = 62)*

Participant no.	Zika virus PRNT titer	DENV1 PRNT titer	DENV2 PRNT titer	Zika virus IgM P/N†	DENV IgM P/N†
1	2,560	10	<10	7.1	1.9
2	640	80	1,280	10.8	1.6
3	2,560	2,560	80	7.0	1.5
4	1,280	5,120	160	7.8	3.5
5	80	<10	<10	7.0	1.2
6	320	<10	<10	1.7	1.1
7	2,560	640	20	4.9	2.6
8	1,280	40	<10	2.4	1.2
9	5,120	<10	<10	2.9	1.2
10	2,560	<10	<10	2.1	0.75
11	1,280	<10	<10	3.5	0.9
12	2,560	2,560	2,560	6.6	2.3
13	640	<10	<10	3.3	2.4
14	320	160	40	3.0	1.6
15	320	160	320	1.9	0.9
16	1,280	20	<10	6.1	0.9
17	320	<10	<10	4.1	1.0
18	320	<10	<10	3.8	1.1
19	1,280	10	<10	4.4	0.92
20	5,120	10	10	1.9	1.0
21	2,560	<10	<10	6.4	0.8
22	2,560	10	<10	6.9	0.9
23	320	1,280	320	2.7	1.1
24	1,280	80	20	10.1	1.8
25	640	<10	<10	10.7	0.9
26	640	<10	<10	3.2	0.6
27	320	<10	<10	7.1	0.7
28	320	160	40	6.2	0.9
29	20,480	80	10	15.2	1.0
30	1,280	10	<10	7.9	0.5
31	640	<10	<10	22.7	1.1
32	1280	20	<10	2.0	0.8
33	2,560	2,560	160	2.0	1.8
34	320	<10	<10	10.1	1.2
35	320	<10	<10	4.1	1.1
36	2,560	10	<10	12.1	1.5
37	80	320	80	12.1	1.2
38	80	320	10	1.6	1.1
39	640	<10	<10	1.8	1.3
40	1,280	10	<10	4.7	1.4
41	640	20	<10	4.9	1.6
42	1,280	1,280	80	3.3	1.0
43	320	<10	<10	6.6	0.7
44	1,280	<10	<10	3.2	1.2
45	640	20	<10	7.1	1.2
46	1,280	320	10	3.8	0.8
47	1,280	2,560	160	4.4	1.0
48	320	1,280	640	3.5	0.7
49	1,280	20	<10	2.4	1.2
50	1,280	2,560	320	3.5	1.2

Participant no.	Zika virus PRNT titer	DENV1 PRNT titer	DENV2 PRNT titer	Zika virus IgM P/N†	DENV IgM P/N†
51	640	<10	<10	2.2	0.86
52	2,560	20	<10	7.5	1.5
53	5,120	40	<10	4.4	0.8
54	2,560	<10	<10	8.8	1.1
55	2,560	40	<10	2.3	1.0
56	20,480	40	<10	2.5	1.1
57	320	<10	<10	4.7	1.7
58	1,280	10	<10	4.0	1.3
59	2,560	2,560	320	7.1	1.0
60	640	<10	<10	6.7	1.5
61	640	<10	<10	2.4	1.4
62	320	640	80	2.6	0.6

* DENV1 = dengue virus serotype 1; DENV2 = dengue virus serotype 2; P/N = positive/negative ratio; PRNT = plaque reduction neutralization test.
†Negative: P/N < 2.0; equivocal: 2 ≤P/N < 3; positive: P/N ≥ 3.
